# Editorial: Genitourinary syndrome of menopause

**DOI:** 10.3389/frph.2022.988943

**Published:** 2022-08-11

**Authors:** José Eleutério Junior, Ana Katherine Silveira Gonçalves

**Affiliations:** ^1^Department of Women's, Child and Adolescent Health, Federal University of Ceará, Fortaleza, Brazil; ^2^Departmento of Obstetrics and Gynecology, Federal University of Rio Grande Do Norte, Natal, Brazil

**Keywords:** female genitourinary disease, menopause, vagina, vulva, therapeutics

With the increase in women's life expectancy, post-menopausal disorders are increasingly studied, and complaints must be effectively addressed based on scientific evidence. In 2014, the term “genitourinary syndrome of menopause” (GSM) was created by the International Society for the Study of Women's Sexual Health and the North American Menopause Society (Sarmento et al.). Worldwide, this condition is still poorly understood by gynecologists. Therefore, studies examining the current concepts and treatments available for GSM are needed, as demonstrated in this special edition of *Frontiers in Reproductive Health*.

GSM comprises a set of symptoms and signs associated with post-menopausal hypoestrogenism. Sarmento et al. showed that this condition is very prevalent, with at least 27% of post-menopausal women meeting the diagnostic criteria for GSM. The genital, sexual, and urinary clinical manifestations seen in GSM are a result of the drop in estrogen levels associated with menopause. This drop in estrogen consequently reduces the vaginal epithelial thickness, vulvar and vaginal lubrication, and epithelial water levels and induces changes in the vaginal microbiome.

Oliveira et al. described the behavior of the vaginal microbiome during the reduction in estrogen levels that accompanies menopause as well as the relationship of the vaginal microbiome with the symptoms presented in GSM. The effective disappearance of lactobacilli results from low glycogen content, since the squamous epithelium of the vagina no longer has an intermediate layer that is rich in glycogen (due to epithelial thinning). Despite the need for further studies on GSM, a relationship between vaginal microbiota (especially in the peri-menopause period) and GSM is apparent.

Therapeutic approaches to GSM include hormone replacement therapy (Costa et al.), the use of vaginal lubricants (Sarmento et al.), microablative radiofrequency therapy (Kamilos et al.), and even the use of laser therapy (Pessoa et al.).

Hormone replacement therapy can entail hormones other than estrogen (as discussed by Costa et al.), despite estrogen being the typical hormone of focus during hormone replacement therapy. Many of the symptoms and effects relating to GSM are associated with hypoestrogenism, leading healthcare workers to consider estrogen replacement alone (either orally or vaginally). In the study by Costa et al., estrogen replacement alone yielded satisfactory results in the treatment of GSM, in most cases. However, the researchers discussed promising alternatives, such as progesterone, testosterone, dehydroepiandrosterone, synthetic steroids, and oxytocin, for hormone replacement therapy in GSM. The authors pointed out that patient situations must be individualized to make the most appropriate choice in the hormonal treatment of GSM.

Some conditions contraindicate the use of hormonal therapy to reduce GSM symptoms. Sarmento et al. reviewed the use of moisturizers and lubricants to treat GSM, considering that vaginal dryness, burning, and consequent dyspareunia are persistent symptoms in GSM that influence the quality of life of women—especially women who cannot undergo hormonal therapy.

Physical, non-hormonal methods have been suggested in the treatment of GSM. Kamilos et al. reviewed the use of microablative fractional radiofrequency (MAFRF) in patients with GSM. MAFRF is a device that uses thermal energy, generated by an electric current, that can be applied to the entire genital area. In clinical trials, MAFRF has improved epithelial trophism (especially in the vagina), influencing the vaginal microbiota and therefore improving symptoms and signs associated with GSM. Therefore, although few studies have examined MAFRF, its results appear promising.

Finally, Pessoa et al. addressed the use of laser therapy through a systematic review and meta-analysis. The authors demonstrated, that although the technique (which is based mainly on a CO_2_ or erbium YAG laser) has been increasingly studied and adopted worldwide, well-designed studies are needed to assess the effectiveness and safety of laser therapy. Questions regarding the effectiveness and safety of laser therapy for GSM have been raised by regulatory bodies such as the Food and Drug Administration (FDA) and the American College of Obstetricians and Gynecologists in the United States.

GSM is a condition that is appearing more frequently in gynecological settings. Understanding the concepts surrounding GSM is essential in making correct diagnoses and treating individual cases with effective therapy and minimal risks.

## Author contributions

All authors listed have made a substantial, direct, and intellectual contribution to the work and approved it for publication.

## Conflict of interest

The authors declare that the research was conducted in the absence of any commercial or financial relationships that could be construed as a potential conflict of interest.

## Publisher's note

All claims expressed in this article are solely those of the authors and do not necessarily represent those of their affiliated organizations, or those of the publisher, the editors and the reviewers. Any product that may be evaluated in this article, or claim that may be made by its manufacturer, is not guaranteed or endorsed by the publisher.

